# K-80003 Inhibition of Macrophage Apoptosis and Necrotic Core Development in Atherosclerotic Vulnerable Plaques

**DOI:** 10.1007/s10557-021-07237-4

**Published:** 2021-08-19

**Authors:** Xiaolei Wang, Zhe Sun, Ruosen Yuan, Weifeng Zhang, Yejiao Shen, Anwen Yin, Yanjie Li, Qingqi Ji, Xia Wang, Yi Li, Min Zhang, Xin Pan, Linghong Shen, Ben He

**Affiliations:** 1 Department of Cardiology, Shanghai Chest Hospital, Shanghai Jiao Tong University, Xuhui Distinct, 241 West Huaihai Road, Shanghai, China; 2 School of Life Science and Technology, Shanghai Tech University, Shanghai, China

**Keywords:** Apoptosis, Macrophage, Autophagy, Oxidative stress, Vulnerable plaque

## Abstract

**Purpose:**

Macrophage apoptosis coupled with a defective phagocytic clearance of the apoptotic cells promotes plaque necrosis in advanced atherosclerosis, which causes acute atherothrombotic vascular disease. Nonsteroidal anti-inflammatory drug sulindac derivative K-80003 treatment was previously reported to dramatically attenuate atherosclerotic plaque progression and destabilization. However, the underlying mechanisms are not fully understood. This study aimed to determine the role of K-80003 on macrophage apoptosis and elucidate the underlying mechanism.

**Methods:**

The mouse model of vulnerable carotid plaque in ApoE^−/−^ mice was developed in vivo. Consequently, mice were randomly grouped into two study groups: the control group and the K-80003 group (30 mg/kg/day). Samples of carotid arteries were collected to determine atherosclerotic necrotic core area, cellular apoptosis, and oxidative stress. The effects of K-80003 on RAW264.7 macrophage apoptosis, oxidative stress, and autophagic flux were also examined in vitro.

**Results:**

K-80003 significantly suppressed necrotic core formation and inhibited cellular apoptosis of vulnerable plaques. K-80003 can also inhibit 7-ketocholesterol-induced macrophage apoptosis in vitro. Furthermore, K-80003 inhibited intraplaque cellular apoptosis mainly through the suppression of oxidative stress, which is a key cause of advanced lesional macrophage apoptosis. Mechanistically, K-80003 prevented 7-ketocholesterol-induced impairment of autophagic flux in macrophages, evidenced by the decreased LC3II and SQSTM1/p62 expression, GFP-RFP-LC3 cancellation upon K-80003 treatment.

**Conclusion:**

Inhibition of macrophage apoptosis and necrotic core formation by autophagy-mediated reduction of oxidative stress is one mechanism of the suppression of plaque progression and destabilization by K-80003.

## Introduction

Cardiovascular disease (CVD) is the leading cause of global mortality, and its incidence is expected to increase in the next few years [1]. Moreover, atherosclerosis is regarded as a chronic disease that is caused by unbalanced lipid metabolism along with inflammation, leading to arterial wall damage which is the basic CVD pathophysiology [2]. Atherosclerotic vulnerable plaque rupture and consequent thrombotic events could result in acute coronary events (e.g., myocardial infarction). Moreover, the apoptosis of macrophages occurs throughout the various stages of atherosclerosis, which serves opposite functions based on the atherosclerotic lesion stage. In advanced atherosclerosis, macrophage apoptosis is a key factor in promoting the formation of a necrotic core, leading to increased instability of atherosclerotic plaques [3, 4]. Therefore, targeting macrophage apoptosis to decrease the formation of necrotic cores could potentially stabilize the atherosclerotic plaques as well as serve as an effective supplement for current treatments [5].

Sulindac sulfide (sulindac hereafter) is among the nonsteroidal anti-inflammatory drugs used to repress the activities of cyclooxygenases (COXs) [6]. A previous study showed that sulindac can also bind to retinoid X receptor-α (RXRα) and induce cancer cell death in an RXRα-dependent manner. A series of sulindac analogs including K-80003 was then synthesized, which can effectively bind to RXRα but cannot inhibit COX-2 activity, because of the increased cardiovascular risk when COX-2 was inhibited [7]. In addition, the anti-atherosclerosis effect of K-80003 was also recently reported aside from the apoptosis induction activity [8]. A mechanistic study suggested that K-80003 administration promoted autophagic flux and further suppressed the NF-κB proinflammatory pathway through an RXRα-dependent mechanism. However, it is unknown whether other mechanisms are also involved in the anti-atherosclerosis K-80003 activity.

Herein, a mouse model of spontaneous vulnerable plaque rupture and cultured murine macrophage cells were utilized to investigate whether K-80003 improve atherosclerotic plaque stability by modulating macrophage apoptosis and elucidate the underlying mechanism. Furthermore, K-80003 was demonstrated to suppress the necrotic core formation of vulnerable plaque by reducing oxidative stress-induced macrophage apoptosis, which was mainly mediated by the promotion of autophagic flux.

## Materials and Methods

### Materials

7-Ketocholesterol, chloroquine, bafilomycin A1, 3-(4, 5)-dimethylthiahiazo (-2-y1)-2, 5-diphenyl-tetrazolium bromide (MTT), dihydroethidine (DHE), and dimethyl sulfoxide (DMSO) were obtained from Sigma-Aldrich (St. Louis, MO, USA). In addition, Lipofectamine 2000 was obtained from Invitrogen (Carlsbad, CA, USA).

### Animal Experiments

Female ApoE^−/−^ mice on a C57BL/6 background were obtained from Jackson Laboratory (Bar Harbor, ME, USA). The mice were kept in a specific pathogen-free environment at 18 °C–22 °C on a 12:12-h light/dark cycle. Additionally, they freely accessed tap water along with a standard laboratory diet. Partial ligation of the left renal artery was performed at 8 weeks old combined with the left common carotid artery (LCCA) as previously described [9]. Briefly, mice were anesthetized using ketamine (100 mg/kg, intraperitoneally) and xylazine (10 mg/kg, intraperitoneally) and maintained at 37 °C on a heating panel. After blunt dissection to expose LCCA and its distal branches. All branches of LCCA except for the left thyroid artery were ligated (6–0 silk) to reduce blood flow. After 1 week, the partial left renal artery surgery was carried out as described previously. Mice were anesthetized, the left renal artery was tied off (6–0 silk) along with a pin gauge (outer diameter = 0.12 mm) after isolation by blunt dissection. Subsequently, the pin gauge was taken out to leave a tight stenosis in artery. The pin gauge was commercially available (Jining Hengsheng Precise Measuring and Cutting Co Ltd, Shandong, China), and its size was verified using a micrometer caliper. Afterward, mice were randomly grouped into two study groups: the control (*n* = 10) and the K-80003 (*n* = 10) groups. Moreover, K-80003 was dissolved in Tween 80, followed by oral gavage administration at 30 mg/kg/day after surgery for eight consecutive weeks. The mice in the control group were administered with an equivalent Tween 80 volume. Experimental mice were sacrificed 8 weeks after surgery.

The Committee on the Medical Ethics of Animal Experiments of the School of Medicine, Shanghai Jiaotong University, approved all the study protocols.

### Cell Growth

The Institute of Biochemistry and Cell Biochemistry and Cell Biology (Shanghai, China) provided all the murine macrophage RAW264.7 cells. Cells were subsequently cultured in Dulbecco’s Modified Eagle Media (HyClone, Logan, UT, USA) enriched with 10% fetal bovine serum (FBS; Beit Haemek, Biological Industries, Israel) along with antibiotics (100 U/mL penicillin as well as streptomycin) in a humidified 5% CO_2_ environment at 37 °C. Cells were collected and subjected to subsequent experiments after different treatments.

### Collection of Tissues and Processing

Isotonic saline was employed to perfuse the mice, followed by isolation by blunt dissection of the left carotid arteries. Tissues were washed with saline solution and then embedded in optimal cutting temperature compound (Sakura Finetechnical Co. Ltd., Tokyo, Japan). Optical coherence tomography (OCT)-embedded segments (5 μm) were cut with a cryotome (FSE, Thermo Scientific, Rockford, IL, USA).

### Histological Staining

Hematoxylin and eosin (H&E) were employed to stain the OCT-embedded left atrial appendage closure (LAAC) segments according to standard protocol. All images were recorded by a light microscope (Leica DM2500, Wetzlar, Germany), and necrotic core area were analyzed using Image-Pro Plus software (Media Cybernetics, Rockville, MD, USA). The areas free of H&E staining were defined as the necrotic core areas.

### Immunofluorescence Staining

OCT-embedded LAAC sections were rinsed by phosphate-buffered saline (PBS) and fixed in 4% cold paraformaldehyde for 20 min, followed by permeabilization for 10 min using 0.2% Triton X-100. Thereafter, blocking of the sections for 30 min was performed using 5% FBS (Life Technologies). Sections were inoculated with primary antibody against cleaved caspase-3 (Ab2302, Abcam, 1:100) at 4 °C overnight incubation, rinsed thrice using PBS, and incubated for 60 min with anti-rabbit IgG/Alexa Fluor 488 antibody (A-21206, Invitrogen, 1:300) performed at room temperature. Consequently, staining with 4′,6-diamidino-2-phenylindole (DAPI; Beyotime Biotechnology, Shanghai, China) was performed for 8 min to visualize the nuclei. Subsequently, a confocal microscope (LSM 710, Zeiss, Jena, Germany) was employed to acquire digital images. Moreover, the Image-Pro Plus software (Media Cybernetics, Rockville, MD, USA) was employed to assess the integrated optical density of the cleaved caspase-3-stained cells. The data are indicated as the percentage of cleaved caspase-3-positive cells.

After different treatments, 4% paraformaldehyde was utilized to fix the RAW264.7 macrophage cells for 15 min at room temperature, and permeabilization with 0.1% Triton X-100 was then performed for 10 min. Furthermore, 5% of bovine serum albumin was employed to block the cells for 1 h. Anti-LC3-II antibody (Ab48394, Abcam, 1:100) were added to the cells for 3 h. The successive steps were similar to the aforementioned protocol.

### Terminal Deoxynucleotidyl Transferase-Mediated dUTP Nick End-Labeling Assay

An in situ cell death detection kit (Roche, Basel, Switzerland) was employed to perform the terminal deoxynucleotidyl transferase-mediated dUTP nick end-labeling (TUNEL) staining. Consequently, 4% paraformaldehyde was used to fix the LCCA segments for 20 min, followed by permeabilization for 2 min on ice using 0.1% Triton X-100. The incubation of the sections with the reaction mixture at 37 °C was performed for 60 min. Furthermore, DAPI was employed to stain the nuclei for 5 min. Thereafter, a confocal laser scanning microscope system (Zeiss LSM 710, Zeiss) was utilized to acquire the images.

### Reactive Oxygen Species Measurement

DHE staining was employed to detect reactive oxygen species (ROS) quantities in LCCA sections. Incubation of the frozen segments with DHE (2 μmol/L) at 37 °C was performed for 20 min, and staining with DAPI was conducted for 5 min. A confocal microscope (Zeiss LSM 710) was employed to capture the images.

Intracellular ROS measures were explored with the ROS assay kit that sets DCFH-DA (Beyotime Biotechnology) as a probe. Moreover, the RAW264.7 macrophage cells that underwent different treatments were inoculated with DCFH-DA-containing serum-free medium and incubated for 20 min at 37 °C, then washed thrice with PBS. The intracellular production of ROS was quantified with a flow cytometer (FACS Calibur, BD Bioscience, Franklin Lakes, NJ, USA) or spectrophotometer (Thermo Fisher, Vantaa, Finland).

### MTT Assays

RAW264.7 macrophage cells were inoculated in 96-well plates, incubated overnight, and treated with varying levels of 7-ketocholesterol (7-KC) or K-80003 or cotreatment. After incubation for 18 h, MTT solution (5 mg/mL) was added post-4 h incubation at 37 °C in the dark. Consequently, 100 μL DMSO was added to solubilize the precipitated formazan, and the determination of the optical density values was performed using a spectrophotometer (Thermo Fisher) at 490 nm.

### Flow Cytometry

Death cells were assessed with an Annexin V-PE Apoptosis Detection Kit (BD Bioscience). Consequently, RAW264.7 macrophage cells were spun at 2,000 rpm for 5 min, and the supernatant was discarded. Cells were then rinsed twice using the Binding Buffer. Moreover, cells were incubated with 100 μL Binding Buffer containing 3% Annexin V staining at 4 °C for 30 min in the dark, after which cells were stained with 5 μL propidium iodide (PI; 50 μg/mL) for 5 min. The cell suspensions were immediately analyzed on a flow cytometer (FACS Calibur, BD Bioscience). Data were evaluated with FlowJo 7.6.1 (Tree Star, Inc., Ashland, OR, USA).

### Western Blot Analysis

Cell lysates were prepared with phenylmethylsulfonyl fluoride (Roche, Boston, MA, USA). The concentrations of the proteins were determined by a BCA Protein Assay Kit (Pierce, Appleton, WI, USA). In addition, the protein was fractionated by 8–12.5% sodium dodecyl sulfate polyacrylamide gel electrophoresis (SDS-PAGE) and then transfer-embedded onto polyvinylidene difluoride membranes (Roche, Mannheim, Germany). Thereafter, 5% skimmed milk in Tris-buffered saline-Tween 20 (TBST) was employed to block the membranes for 1 h at room temperature; the membrane was incubated with appropriate primary antibodies against PARP (#9532, CST, 1:1000), cleaved caspase-3 (#9661, CST, 1:1000), SOD2 (#1314, CST, 1:1000), LC-3I/II (Ab128025, Abcam, 1:1000), p62 (#8025, CST, 1:1000), and β-actin (Ab6276, Abcam, 1:5000); and incubated overnight at 4 °C. Furthermore, the membranes were rinsed thrice using TBST and then inoculated with horseradish peroxidase-linked secondary antibodies and incubated for 1 h at room temperature. The protein was captured with Image Quant LAS 4000 Imager (GE, Boston, MA, USA). Consequently, the quantification of the intensity of the protein bands was assessed with a Gel-Pro analyzer. β-actin served as the normalization standard.

### mGFP-RFP-LC3 Transfection and Fluorescent LC3 Puncta Assessment

RAW264.7 macrophage cells were transiently transfected with the mGFP-RFP-LC3 adenovirus via Lipofectamine 2000 (Invitrogen) as per the manufacturer-provided protocol. After designated treatments, 4% PFA was employed to fix the cells for 15 min. Thereafter, the nuclei were stained using DAPI for 5 min. LC3 puncta were observed using confocal microscopy (LSM 710, Zeiss). In the autophagic cell quantifications, determination of the GFP-LC3 and mRFP-LC3 punctuated dots was performed from at least three independent visual fields by counting more than 30 cells. Red and Green Puncta Colocalization Macro were employed to quantify LC3 puncta with the Image J program as documented [10]. GFP + /RFP + puncta constitute the yellow dots (autophagosomes), and GFP − /RFP + puncta comprise the red dots (autolysosomes). Experiments were conducted in triplicates.

### Transmission Electron Microscopy

After different treatments, 2% glutaraldehyde was employed to fix the RAW264.7 macrophages at 4 °C overnight, postfixed in 1% osmium tetroxide for 2 h, and dehydration in graded alcohols. The macrophages were then embedded in Epo resin. A CM-120 electron microscope (Philips, Amsterdam, Netherlands) was employed to assess the autophagosomes and autolysosomes in cells.

### Statistical Analysis

Values were expressed as mean ± SEM and analyzed using the Statistical Package for the Social Sciences, version 18.0 (IBM, Chicago, IL, USA). Student’s *t*-test was employed to compare the data between the two groups. Moreover, comparisons among groups were made using one-way analysis of variance, followed by Tukey’s test for multiple comparisons. *P* < 0.05 indicated statistical significance.

## Results

1. K-80003 Attenuates Necrotic Core and Cellular Apoptosis of Vulnerable Plaques in ApoE^−/−^ Mice

**Fig. 1:**
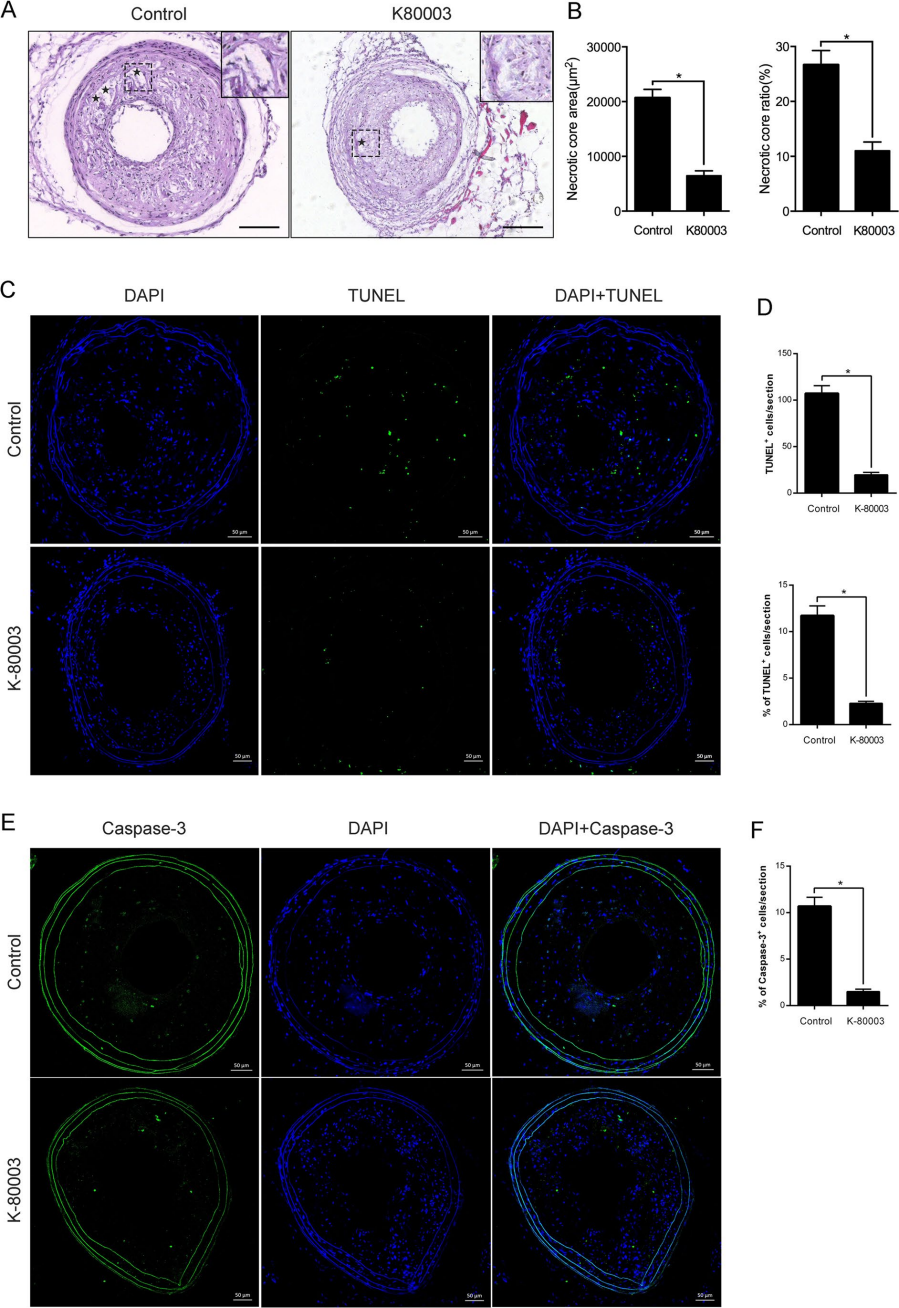
Effects of K-80003 on necrotic core and cellular apoptosis of atherosclerotic plaques in ApoE^−/−^ mice. **A** H&E staining in the left carotid arteries lesions from the control and K-80003-treated ApoE^−/−^ mice (*shaded star* necrotic areas) and **B** quantification of the necrotic core area (*left*) and ratio (*right*); *n* = 5 animals per group, scale = 100 μm, *P < 0.05). **C** TUNEL staining was used to detect the number and the proportion of TUNEL-positive cells in the control and K-80003 group; **E** immunofluorescence staining was used to determine the number of cleaved caspase-3 cells (apoptosis) in atherosclerotic plaques of the control and K-80003 groups (*n* = 5 animals per group), scale = 50 μm, *P < 0.05). **D**, **F** Quantification of TUNEL + and cleaved caspase-3 + cells (*n* = 5 animals per group)

Typical vulnerable plaques are linked to highly inflammatory cell levels as well as a large necrotic core covered by a thin fibrous cap [11]. A mouse model of a vulnerable atherosclerotic plaque was created to investigate the effects of K-80003 on plaque necrosis and cellular apoptosis. K-80003 significantly reduced the necrotic core absolute area and the proportion of the necrotic core in the plaque area compared with the vehicle group (6,466 ± 893 μm^2^ vs. 20,668 ± 1,546 μm^2^, *P* < 0.05; 11.1% ± 1.6% vs. 26.7% ± 3.3%, *P* < 0.05; Fig. 1A and B). Moreover, the anti-apoptotic impacts of K-80003 were then explored by examining the portion of apoptotic cells via the TUNEL assay (Fig. 1C). The results of this study showed that the number [19.7 ± 2.6 vs. 107.3 ± 8.2, *P* < 0.05; Fig. 1D (above)] and proportion of apoptotic cells [1.7% ± 0.3% vs. 10.7% ± 1.0%, *P* < 0.05; Fig. 1D (below)] in the lesions from K-80003-treated mice were remarkably lower compared with those in the control group. Furthermore, a remarkable decline exists in the proportion of cells with cleaved caspase-3-positive cells in the lesions of the K-80003 group compared with the control mice (1.7% ± 0.3% vs. 10.7% ± 1.0%, *P* < 0.05; Fig. 1E and F). Thus, these data suggested that K-80003 could reduce the plaque necrosis formation and suppress cellular apoptosis in vulnerable plaques in ApoE^−/−^ mice.

2. K-80003 Inhibits 7-KC-Triggered Macrophage Apoptosis In Vitro

**Fig. 2:**
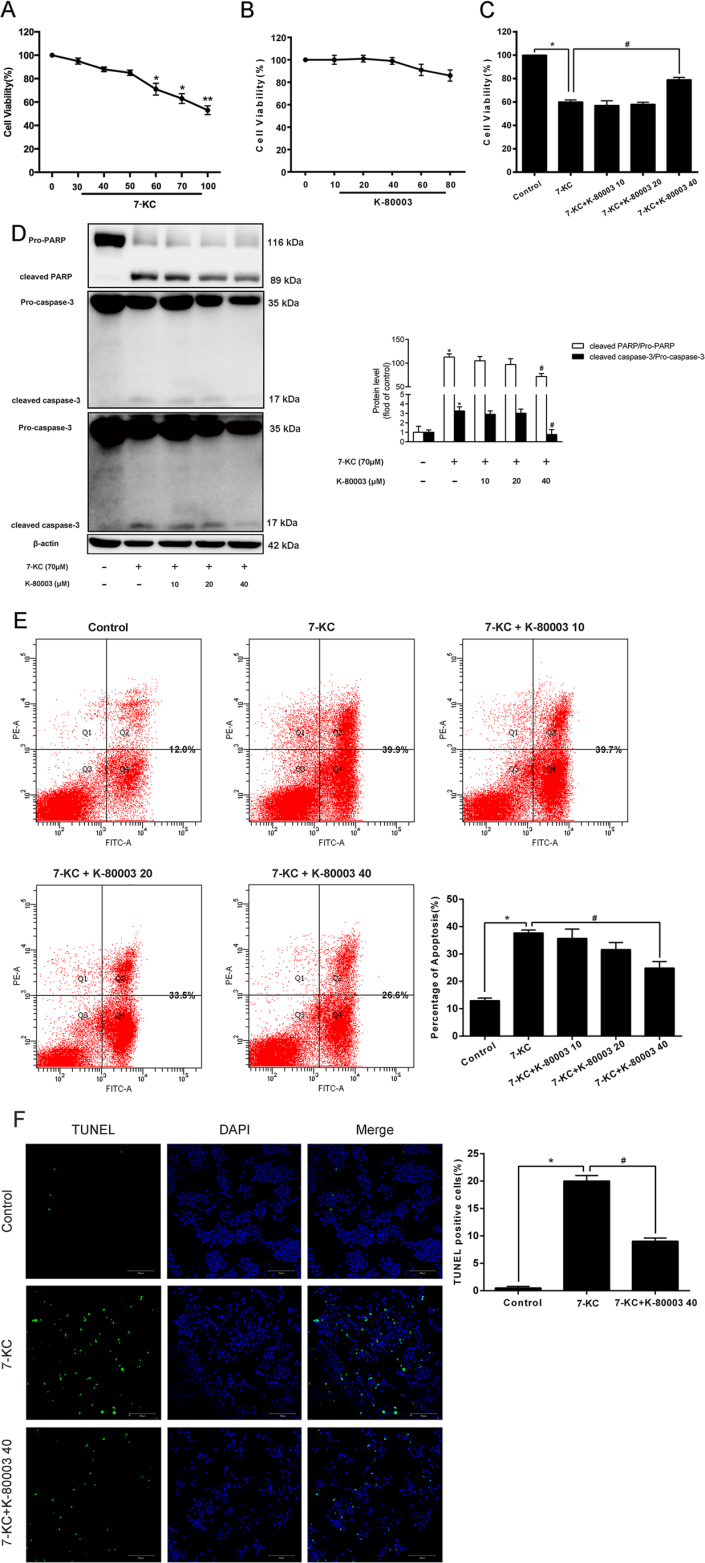
Effect of K-80003 on 7-KC induced murine RAW264.7 macrophage apoptosis. **A**, **B** Murine RAW 264.7 cells were incubated in varying doses of 7-KC (0, 30, 40, 50, 60, 70, and 100 μM) or K-80003 (0, 10, 20, 40, 60, and 80 μM) for 18 h, and cell viability was assessed by MTT assay. RAW 264.7 cells were cotreated with 70 μmol/L 7-KC and different concentrations of K-80003 (0, 10, 20, and 40 μM) for 18 h compared with control. Cell viability was assessed by MTT assay (**C**), western blotting was used to detect cleaved PARP/pro-PARP and cleaved caspase-3/pro-caspase-3 expression (**D**), and cells were labeled with annexin V and PI staining with flow cytometry (**E**). **F** Murine RAW 264.7 cells were treated with 7-KC (70 μM) and K-80003 (40 μM) for 18 h, representative images of TUNEL staining of macrophages showed the apoptotic cells (apoptotic cells stained in *green* and nucleus stained in *blue* with DAPI). The number of TUNEL-positive cells was measured and quantitated. *P < 0.05 compared with the control group and ^*#*^*P* < 0.05 compared to cells treated with 7-KC. Data are presented as mean ± SEM of three independent experiments

Whether K-80003 suppressed apoptosis in RAW264.7 macrophages was next examined. 7-KC is among the oxysterols in ox-low-density lipoprotein (LDL) that promote oxidative stress and apoptosis in macrophages [12, 13]. Thus, treatment was done with diverse 7-KC concentrations (0, 30, 40, 50, 60, 70, and 100 μmol/L) for 18 h. Consequently, a dose-dependent approach was reported with the 7-KC treatment. The results showed that 70 μM was the ideal concentration for inducing apoptosis and reducing cell viability by 37% (Fig. 2A). Moreover, the effect of K-80003 on the viability of the RAW264.7 cells was then tested after administration with various levels (0, 10, 20, 40, 60, and 80 μM) of K-80003 for 18 h. However, no significant differences were found in the viability of the cells among the groups (Fig. 2B, P > 0.05). The data demonstrated that these K-80003 concentrations did not affect the viability of the RAW264.7 cells. Cells with 7-KC and K-80003 were treated next for 18 h. In addition, K-80003 treatment significantly inhibited 7-KC-triggered macrophage apoptosis in a dose-dependent manner as evidenced by the increasing viability of the cells (Fig. 2C), downregulating the cleaved PARP/pro-PARP expression as well as cleaved caspase-3/pro-caspase-3 (Fig. 2D), and decreasing apoptosis conforming to the staining with Annexin V/ PI (Fig. 2E). Consequently, this finding was further supported by the TUNEL assay, which revealed that the percentage of TUNEL-positive cells was remarkably decreased (Fig. 2F). Altogether, these data suggested that administration of K-80003 protected against macrophage death in vitro.

3. K-80003 Prevents Macrophage Apoptosis by Ameliorating Oxidative Stress

**Fig. 3:**
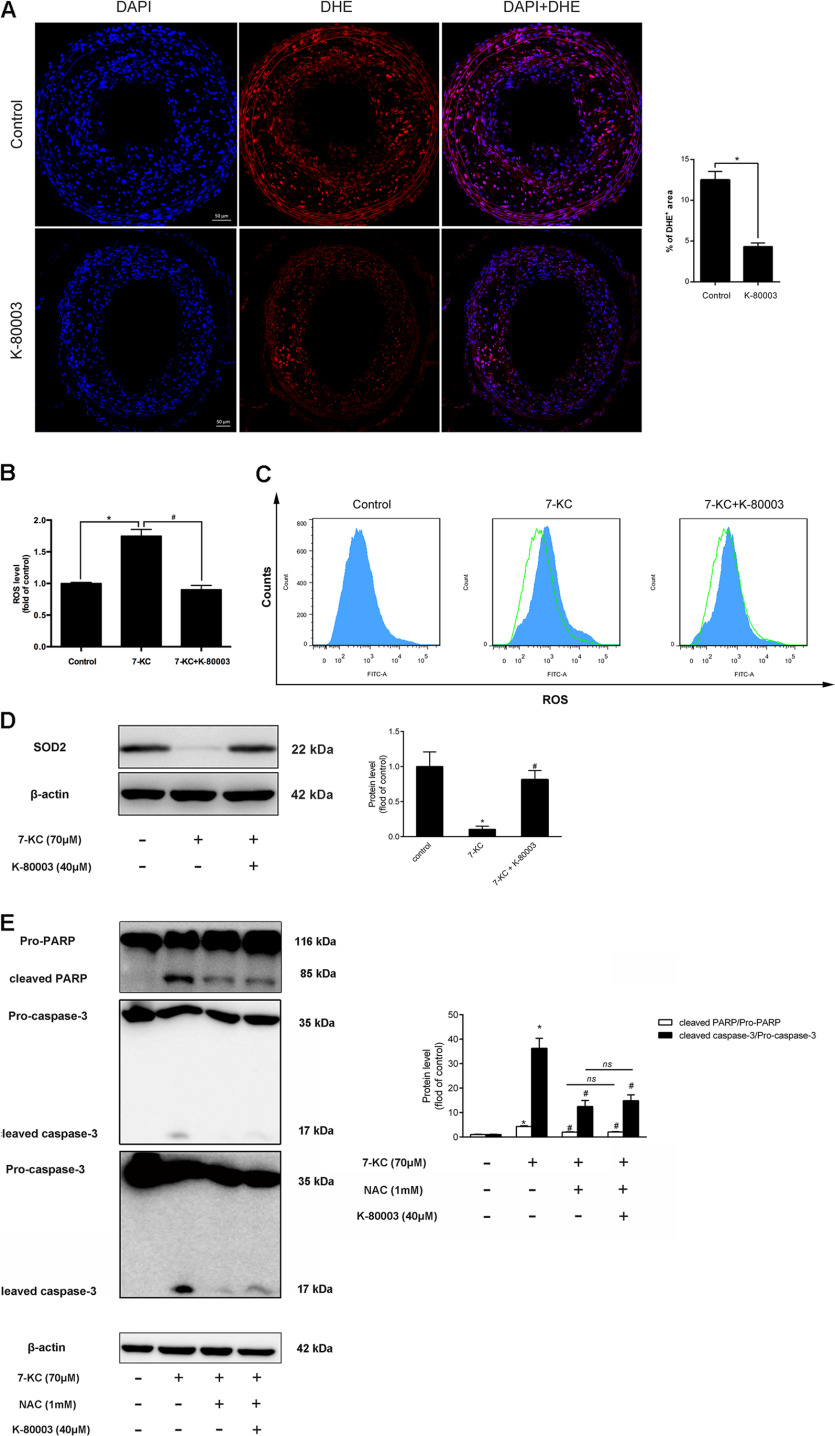
K-80003 prevents RAW264.7 macrophage apoptosis by ameliorating oxidative stress. **A** DHE staining of lesions from ApoE^−/−^ control and K-80003-treated mice and quantification of relative fluorescence intensity (*n* = 5 animals per group, scale = 50 μm, *P < 0.05). RAW264.7 cells were treated with 7-KC (70 μM) with or without K-80003(40 μM) for 18 h, and fluorescent label probe DCFH-DA was used to detect intracellular ROS production. Fluorescence formation was quantified by spectrophotometer (**B**) or flow cytometry (**C**). Western blotting was used to detect SOD2 expression (**D**). **E** RAW 264.7 cells were treated with 7-KC (70 μM), K-80003 (40 μM), and NAC (1 mM) for 18 h. Western blotting was used to detect cleaved PARP/pro-PARP and cleaved caspase-3/pro-caspase-3 protein. *P < 0.05 compared with the control group; ^*#*^*P* < 0.05 compared with the 7-KC group. Data were presented as mean ± SEM of at least three independent experiments

Oxidative stresses are important causes of macrophage apoptosis in advanced atherosclerotic plaque [14]. Thus, the effect of K-80003 on ROS generation in vulnerable plaques and 7-KC-induced macrophages was further explored. Oxidative stress in the atherosclerotic plaque tissue of ApoE^−/−^ mice was detected by ROS-DHE. The ROS in the K-80003 group was remarkably lower compared with the control group (4.3% ± 0.4% vs. 12.5% ± 1.0%, *P* < 0.05; Fig. 3A). In vitro, this study found that K-80003 could attenuate 7-KC-induced intracellular ROS production (Fig. 3B and C) and upregulate SOD2 (antioxidant enzymes) expression (Fig. 3D). The macrophage cells were administered with ROS scavenger *N*-acetyl-L-cysteine (NAC) to investigate whether K-80003 inhibited apoptosis by ameliorating oxidative stress. However, no significant differences were found in the cleaved PARP/pro-PARP and cleaved caspase-3/pro-caspase-3 expression between the 7-KC + K-80003 + NAC and the 7-KC + NAC groups (Fig. 3E). Therefore, K-80003 prevented macrophage apoptosis by ameliorating oxidative stress.

4. K-80003 Suppresses Oxidative Stress-Induced Apoptosis Through Modulating Autophagy

**Fig. 4:**
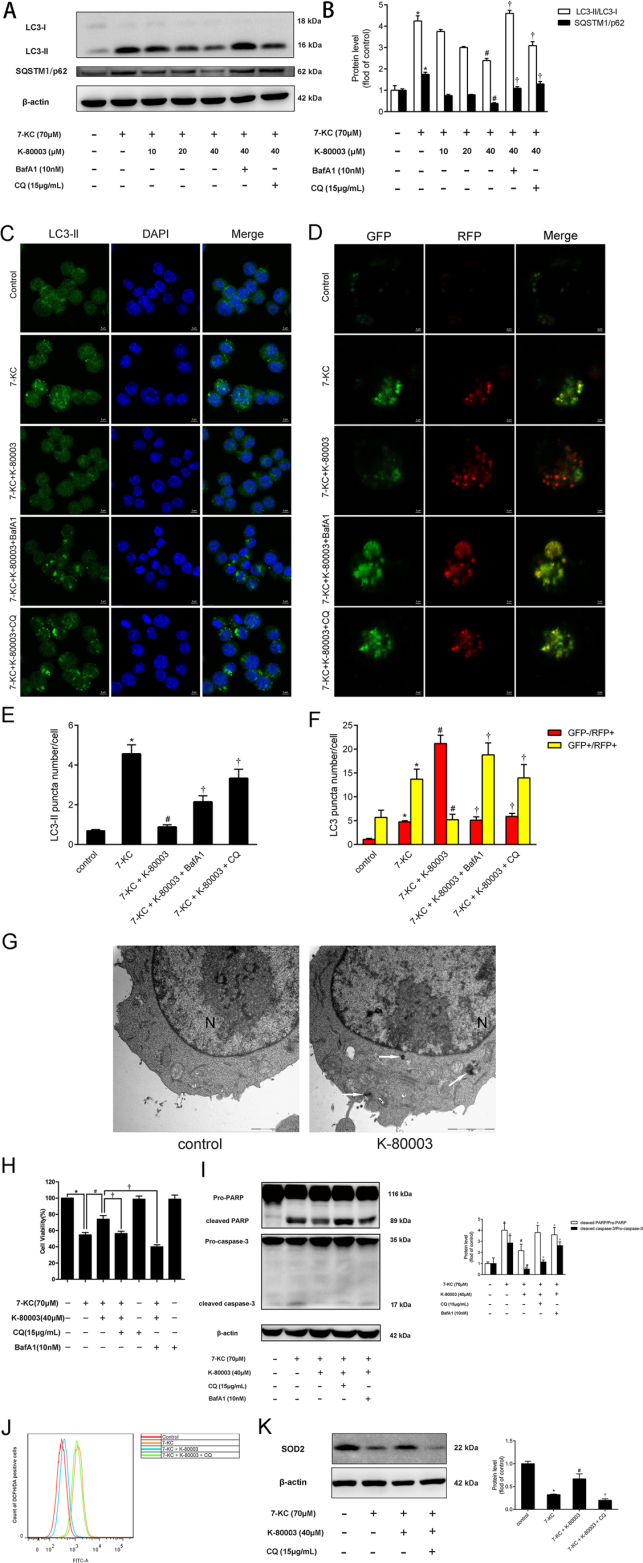
K-80003 suppresses oxidative stress-mediated RAW264.7 macrophage apoptosis through restoring impaired autophagy. **A** RAW264.7 cells were treated with 7-KC (70 μM) with or without different concentrations of K-80003 (10, 20, and 40 μM) for 18 h, and BafA1 (10 nM) or CQ (15 μg/mL) was added to the cultures for the final 4 h. The expression of autophagy markers (LC3I, LC3II, and SQSTM1/p62) were detected by western blotting. **B** Histograms show the quantitative analysis of LC3 and SQSTM1/p62. **C** Cells were stimulated as prescribed above, cells were stained by LC3II (*green*) and DAPI (*blue*) and detected by confocal microscopy; scale bar = 5 μm. **D** Representative confocal microscopy images and quantification of autophagosomes (*yellow dots* generated from the overlap of GFP and RFP puncta) and autolysosomes (*red dots* generated from RFP puncta) in RAW264.7 cells transfected with the RFP-GFP-adenovirus; scale bar = 2 μm. **E** GFP-LC3II puncta number per cell was quantified using the ImageJ program. **F** Percentages of cells with puncta like LC3 were figured up based on the images represented in **D**, dividing into GFP + /RFP + group (*yellow column*) and GFP − /RFP + group (*red column*). **G** Representative transmission electron microscopy (TEM) images of cells with or without K-80003 for 18 h. *Arrows* indicate autophagosome or autolysosome structures. *N* nuclei, scale bar = 2 µm. *P < 0.05 compared with the control group; ^*#*^*P* < 0.05 compared with the 7-KC group; and ^*†*^*P* < 0.05 compared with the 7-KC + K-80003 group. Data were presented as mean ± SEM of at least three independent experiments. **H**, **I** RAW264.7 cells were treated with 7-KC (70 μM) with or without K-80003 (40 μM) for 18 h, and BafA1 (10 nM) or CQ (15 μg/mL) was added to cultures for the final 4 h. Cell viability was assessed by MTT assay (**H**), western blotting was used to detect cleaved PARP/pro-PARP and cleaved caspase-3/pro-caspase-3 expression (**I**). RAW264.7 cells were treated with 7-KC (70 μM) with or without K-80003 (40 μM) for 18 h, CQ (15 μg/mL) was added to cultures for the final 4 h, intracellular ROS production was measured by flow cytometry (**J**), and SOD2 protein was analyzed by Western blotting (**K**). *P < 0.05 compared with the control group, ^*#*^*P* < 0.05 compared with the 7-KC group, and ^*†*^*P* < 0.05 compared with the 7-KC + K-80003 group. Data are presented as mean ± SEM of three independent experiments

Studies have shown that 7-KC can block autophagy flux. Figure 4A shows that the administration of 7-KC markedly raised the LC3II/LC3I ratio as well as the SQSTM1/p62 expression, suggesting that 7-KC blocked the autophagy flux of macrophages. Surprisingly, K-80003 downregulated the LC3II/LC3I ratio as well as SQSTM1/p62 in macrophages induced with 7-KC. In addition, this effect could be blocked by two specific inhibitors of autophagy, bafilomycin A1 (BafA1) or chloroquine (CQ), indicating that K-80003 rescued 7-KC-induced autophagy dysfunction. Furthermore, LC3II immunofluorescence staining, a marker of autophagic vesicle which is eventually degraded in the lysosome, significantly increased compared with the control group after 7-KC treatment, and K-80003 significantly downregulated LC3II compared with the 7-KC group (Fig. 4C), which could also be reversed by BafA1 or CQ. A kind of mGFP-RFP-LC3 adenovirus was used to further examine the autophagic flux process [15]*.* Moreover, the autophagosomes were labeled with yellow dots (merging of green and red puncta), while the autolysosomes were labeled with free red dots. Figure 4D shows that the puncta in 7-KC-treated cells appeared to become yellow (GFP + /RFP +), whereas those in the K-80003 group appeared to be red (GFP − /RFP +) indicating that K-80003 promoted autophagic flux. Likewise, BafA1 and CQ markedly blunted the effects of K-80003 on the red puncta promotion. Transmission electron microscopy was also employed to assess the K-80003-triggered morphological alterations. Furthermore, K-80003-treated cells demonstrated cytoplasmic accumulation of autophagosomes and autolysosomes, morphological markers of autophagy, after K-80003 treatment for 18 h (Fig. 4G). These findings indicated that K-80003 could improve the impaired autophagic flux in 7-KC-induced macrophages.

RAW264.7 macrophage cells were exposed to the autophagy inhibitors BafA1 or CQ for the final 4 h in K-80003 presence (40 μM) followed by the 7-KC stimulation to test whether the anti-apoptotic and anti-oxidative effects of K-80003 were dependent on autophagy. Figure 4H showed that the K-80003 effect in promoting cell viability was reversed by the addition of BafA1 or CQ. Moreover, higher expression of cleaved caspase-3/pro-caspase-3, as well as cleaved PARP/pro-PARP, was found after blocking autophagy (Fig. 4I). Moreover, the cotreatment of K-80003 and autophagy suppressors caused a remarkable increase in ROS quantities and decreased SOD2 expression in RAW264.7 cells (Fig. 4J, K). Therefore, these results proved that the protective function of the apoptosis reaction of K-80003 is dependent on promoting autophagy efflux.

## Discussion

The present study found that K-80003 treatment could improve atherosclerotic plaque stability by inhibiting cellular apoptosis and consequently reducing necrotic core. Mechanistically, K-80003 prevented 7-KC triggered macrophage apoptosis by autophagy-mediated reduction of oxidative stress.

Destabilization of atherosclerotic plaque along with the rupture with thrombosis constitute a pivotal pathological mechanism responsible for adverse cardiovascular events consisting of cardiovascular death as well as acute myocardial infarction [16]. Potent prevention particularly targeting plaque stabilization remains to be explored despite the evolving anti-inflammatory as well as lipid-lowering therapies that can reduce acute coronary syndrome. An essential goal of reducing the prevalence of deadly coronary events includes promoting plaque stabilization. The characteristics of a vulnerable plaque include a huge necrotic core that is covered by a thin fibrous cap, inflammation, and, sometimes, intraplaque hemorrhage. Macrophage apoptosis serves opposite functions in plaque progression: enhanced macrophage apoptosis along with efferocytosis (phagocytic clearance of death cells) reduces early atheroma burden while macrophage apoptosis, accompanied with defective efferocytosis, facilitates the expansion of the necrotic core and consequently leads to accelerating atherosclerosis and even plaque rupture in advanced lesions [3, 4, 17, 18]. In recent years, many molecular imaging experimental methods are designed for detecting apoptotic cells, including positron emission tomography and single-photon emission computed tomography [19, 20]. These methods are developed to identify tumor cell death. Nevertheless, they also hold the potential for molecular imaging of apoptotic cells to determine vulnerable plaques [21]. The current treatments for preventing acute coronary events consist of statin drugs that improve the stability of vulnerable plaques by its pleiotropic effects (e.g., inhibiting inflammation) [22, 23]. A growing number of studies have documented the proprotein convertase subtilisin-kexin type 9 (PCSK9) that causes LDL receptor protein degradation as a novel therapeutic target for atherosclerosis prevention and treatment [24]. Monoclonal PCSK9 antibodies, which block the impacts of PCSK9 on LDL receptors indicated beneficial results in cardiovascular outcome trials [25]. In addition, PCSK9 is upregulated in ox-LDL-triggered human umbilical vein endothelial cells and mediates apoptosis [25]. PCSK9 is also upregulated by ox-LDL in macrophage, which seems to participate in inflammatory signaling, suggesting potential roles for PCSK9 in mediating inflammation as well as apoptosis in atherosclerotic plaques.

Through the constructed mouse model of spontaneous plaque rupture, our previous study found that K-80003 repressed atherosclerotic plaque progression as well as destabilization via inhibition of the NF-κB proinflammatory pathway [8]. The NF-κB signaling pathway plays important roles in inflammatory, cell cycle and apoptosis [26]. An early study demonstrated that activation of NF-κB reduced cell apoptosis in cell [27], whereas most studies showed that NF-κB is a pro-apoptotic transcription factor correlated with cell injury [28, 29]. A previous study showed that K-80003 exerts anti-cancer effect by inducing TNFα-dependent apoptosis [7]. Due to the key role of macrophage apoptosis in vulnerable plaque formation and the complexity of NF-κB signaling pathway in regulating cellular apoptosis, it is significant to determine the impact of K-80003 on macrophage apoptosis. Our results demonstrated that K-80003 treatment enhanced the stability of vulnerable atherosclerotic plaques by reducing the plaque necrotic core and cellular apoptosis. In vitro, K-80003 can also inhibit 7-KC-triggered macrophage apoptosis, thus providing a new strategy for stabilizing atherosclerotic plaque. Furthermore, the prospective mechanism of the ability of inhibition macrophage apoptosis by K-80003 was assessed.

Autophagy, being widespread in eukaryotic cells, refers to cells stimulated by external stimuli (e.g., nutritional deficiencies, hypoxia or oxidative stress, and so on) where the intracellular material is wrapped by the double-membrane structure and transported to the lysosome. Ultimately, hydrolytic enzymes degrade the aging organelles or misfolded proteins and are recycled or used to provide energy. The whole process is referred to as autophagy flux [30]. In vitro studies uncovered several promising triggers of autophagy that are present in atherosclerotic plaques, including the production of reactive oxygen species and aggregation of oxidized lipoproteins [31, 32]. Autophagy attenuates oxidative stress and decreases cell apoptosis as well as protects against atherosclerotic plaque instability (e.g., defective efferocytosis as well as large necrotic cores) [33–35]. However, mounting evidence showed the defect of autophagic flux in advanced vulnerable plaques. Consequently, Razani et al. [14] documented that autophagy is defective with plaque progression which enhances atherosclerosis. Several lines of evidence indicated that suppressing autophagy in the atherosclerotic environment exacerbated atherosclerosis lesion progression in both animal models [36] and human carotid tissue [37]. However, upregulation of autophagy can regulate the progression of atherosclerosis as well as reduce the vulnerability of atherosclerotic plaques [38]. Thus, promoting macrophage autophagy may present a novel therapeutic approach to stabilize vulnerable plaque. The findings herein provided convincing pieces of evidence that K-80003 restored 7-KC-induced impaired autophagy flux in murine macrophage and consequently reduced oxidative stress-induced apoptosis. Nonetheless, some molecular mechanisms that need further investigations still exist.

**Fig. 5:**
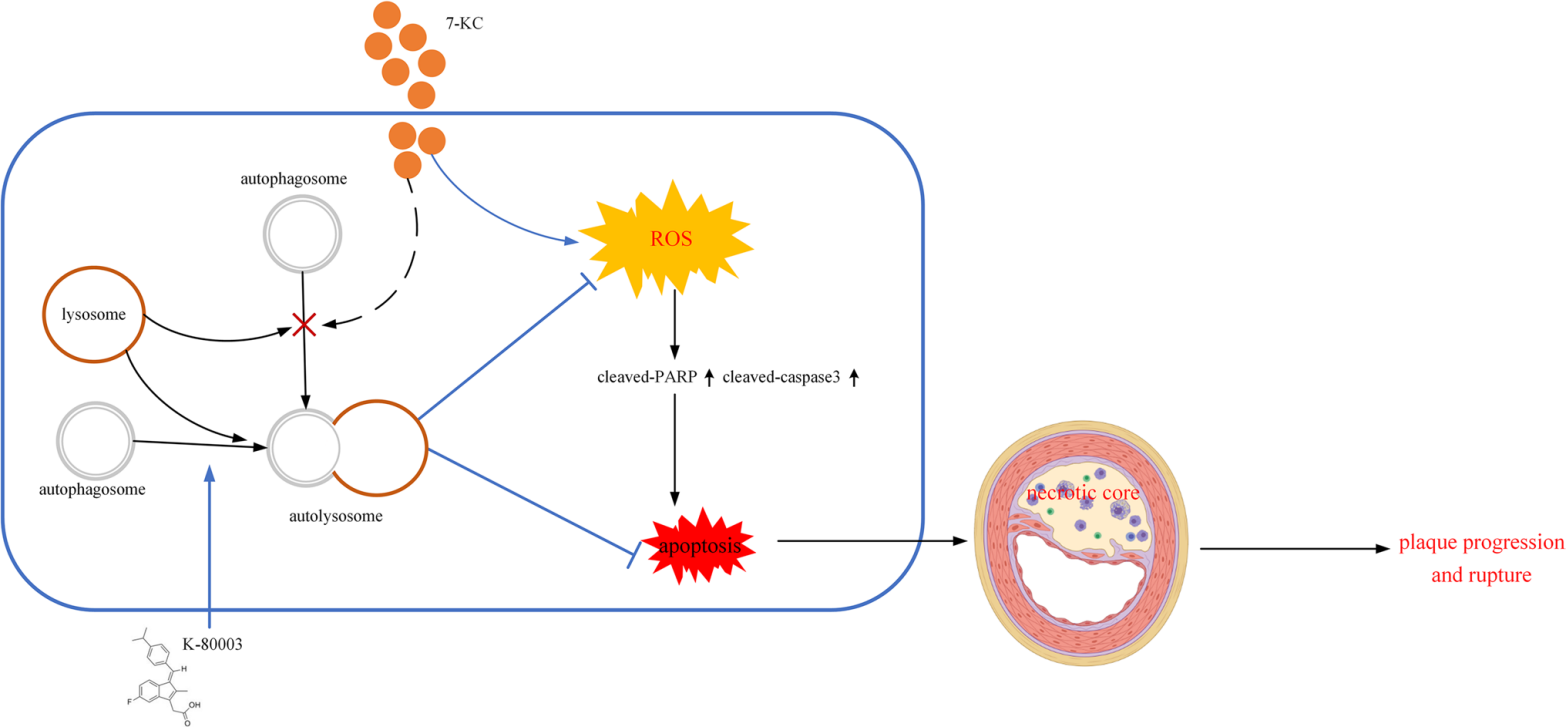
Graphic summary of the effects of K-80003 on autophagy, oxidative stress, apoptosis, and atherosclerosis progression. When exposed to excessive 7-KC, macrophage autophagy flux is blocked accompanied with accumulated oxidative stress. K-80003 could upregulate autophagic activity to reduce oxidative stress-induced apoptosis and consequently inhibition of the necrotic core formation and plaque progression and rupture

In summary, this study demonstrated that K-80003 can protect against macrophage apoptosis by autophagy-mediated reduction of oxidative stress, which can suppress the necrotic core formation of vulnerable plaque in the carotid artery of AopE^−/−^ mice (Fig. 5). Hence, an alternative therapeutic approach for stabilizing atherosclerotic plaque was provided.

## Data Availability

Not applicable.
